# Pyrroloquinoline Quinone Improves Ram Sperm Quality through Its Antioxidative Ability during Storage at 4 °C

**DOI:** 10.3390/antiox13010104

**Published:** 2024-01-15

**Authors:** Zhendong Zhu, Wenjia Li, Qitai Yang, Haolong Zhao, Weijing Zhang, Adedeji O. Adetunji, S. A. Masudul Hoque, Xin Kou, Lingjiang Min

**Affiliations:** 1 College of Animal Science and Technology, Qingdao Agricultural University, No. 700 Changcheng Road, Qingdao 266109, China; zzd2020@qau.edu.cn (Z.Z.);; 2 Department of Agriculture, University of Arkansas at Pine Bluff, Pine Bluff, AR 71601, USA; 3 Department of Animal Breeding and Genetics, Bangabandhu Sheikh Mujibur Rahman Agricultural University, Gazipur 1706, Bangladesh; 4 Hongde Livestock Farm, Yingli Town, Weifang 261000, China

**Keywords:** mitochondria, PQQ, quality, ROS, ram sperm

## Abstract

Sperm motility is an important factor in the migration of sperm from the uterus to the oviduct. During sperm preservation in vitro, sperm generates excessive ROS that damages its function. This study aims to investigate whether the addition of pyrroloquinoline quinone (PQQ) to the diluted medium could improve chilled ram sperm quality, and then elucidates the mechanism. Ram semen was diluted with Tris-citric acid-glucose (TCG) medium containing different doses of PQQ (0 nM, 10 nM, 100 nM, 1000 nM, 10,000 nM), and stored at 4 °C. Sperm motility patterns, plasma membrane integrity, acrosome integrity, mitochondrial membrane potential, reactive oxygen species (ROS) levels, malondialdehyde (MDA) levels, superoxide dismutase (SOD) activity, and ATP levels were measured after preservation. Furthermore, the expressions of NADH dehydrogenase 1 (MT-ND1) and NADH dehydrogenase 6 (MT-ND6) in sperm were also detected by western blotting. In addition, sperm capacitation and the ability of sperm to bind to the zona pellucina were also evaluated. It was observed that the addition of PQQ significantly (*p* < 0.05) improved ram sperm motility, membrane integrity, and acrosome integrity during preservation. The percentage of sperm with high mitochondrial membrane potential in the PQQ treatment group was much higher than that in the control. In addition, supplementation of PQQ also decreased the sperm MDA and ROS levels, while increasing ATP levels. Interestingly, the levels of MT-ND1 and MT-ND6 protein in sperm treated with PQQ were also higher than that of the control. Furthermore, the addition of 100 nM PQQ to the medium decreased ROS damage in MT-ND1 and MT-ND6 proteins. The addition of 100 nM PQQ significantly (*p* < 0.05) increased protein tyrosine phosphorylation in ram sperm after induced capacitation. Furthermore, the value of the sperm–zona pellucida binding capacity in the 100 nM PQQ treatment group was also much higher than that of the control. Overall, during chilled ram- sperm preservation, PQQ protected ram sperm quality by quenching the ROS levels to reduce ROS damage and maintain sperm mitochondrial function, and preserved the sperm’s high ability of fertilization.

## 1. Introduction

Artificial insemination (AI) is a modern advanced technology that has been widely used in several animal species around the world. While AI typically involves the use of fresh, cooled, or cryopreserved semen, sheep AI uniquely relies on fresh semen due to challenges associated with cooled or cryopreserved sperm, which include compromised sperm quality and reduced fertility outcomes [1,2]. Despite many technical advances in the past decades, AI in sheep has not achieved the widespread use that has occurred in the cattle or swine industries. This is a result of low economic return, variable fertility rates, and more rapid loss of semen quality with storage time [3]. Compared to other species, ram sperm contains a lower intracellular cholesterol to phospholipid ratio which makes it more sensitive to cold shock [2]. Reducing cold shock by the supplementation of additives to the preserved medium might improve ram sperm quality.

During ram sperm storage at 4 °C, sperm generates a significant amount of reactive oxygen species (ROS) due to the unphysiological storage conditions leading to oxidative stress [3]. Though sperm possesses an antioxidant system that helps reduce ROS levels, the ability of sperm to scavenge ROS is limited because most of the cytoplasm is discarded during spermatogenesis and maturation [4]. Previous studies have shown that excessive ROS damages sperm motility, membrane integrity, acrosome integrity, DNA integrity, and fertilization [4,5,6]. In addition, it has been reported that ROS damages sperm mitochondria [7] structure [7] and changes the concentration of intracellular calcium ions to induce apoptosis [8,9]. In our previous study, when the sperm mitochondrial transcription system was attacked by excessive ROS, it decreased sperm mitochondrial ATP generation [10]. Decreasing the ROS damage by reducing ROS levels is essential to maintain sperm quality during preservation in vitro. Indeed, studies have shown that antioxidants such as ascorbic acid [11], lycopene [12], melatonin [13], resveratrol [14], vitamin E [15], and epimedium [16] have been added to sperm diluents to improve ram sperm quality during storage at 4 °C or cryopreservation. In addition, Buck et al. (2012) [17] found that the addition of 2 and 4 mM methionine, one of the precursors of glutathione, improved ram sperm viability and mitochondrial activity during the 96 h of storage at 5 °C, when compared to the control. Therefore, supplementation of antioxidants to the diluted medium may help in improving ram sperm quality by reducing the ROS damage during ram sperm storage at 4 °C.

Pyrroloquinoline quinone (PQQ), a redox cofactor [18], has been proven to be present in plant products [19], mammalian organs [20], and milk [21]. Additionally, various physiological and medicinal functions of PQQ have so far been reported [22,23,24]. Kumazawa et al. (2007) [25] and Tachaparian et al. (2010) [26] found that PQQ induced mammalian cells’ mitochondrial biogenesis and metabolism by interacting with various cell signaling and upregulating key genes. In addition, PQQ was also found to play an important role in the central and peripheral nervous system. For example, PQQ inhibited 6-hydroxydopamine-induced neurotoxicity [27], increased the astroglial cells’ nerve growth factor level [28], and promoted sciatic nerve generation in rats [29]. Beyond its function in the nervous system, PQQ also plays a role in the reproductive system. When alkylating agents were used to induce ovarian dysfunction in a mice model [30], the addition of PQQ increased the ovarian weight and size, partially normalized the disrupted estrous cycle period, and prevented the loss of follicles. This suggests that PQQ plays a role in promoting the cell proliferation of granulosa cells, inhibiting cell apoptosis of granulosa and cell senescence of ovarian stromal cells. Moreover, in terms of the follicular development process, Hoque et al. (2021) found that ROS-associated damage in FSH-stimulated granulosa cells adversely affected their physiology and follicular health. However, the supplementation of PQQ helped to decrease ROS damage and increased both the number of ovulated oocytes and pups per delivery [31]. In addition, it was observed that the administration of PQQ significantly increased semen quality in aging layer breeder roosters [32]. In our previous study, when boar sperm was under ROS stress stimulated by low glucose conditions, boar sperm mitochondrial ATP generation was decreased as ROS attacked the boar sperm mitochondrial transcription system. In this case, the supplementation of PQQ reduced the mitochondrial damage and increased the boar sperm linear motility by enhancing mitochondrial ATP generation [33]. On this note, the present study aimed to investigate the role and mechanism by which PQQ may improve ram sperm quality during storage at 4 °C.

## 2. Materials and Methods

### 2.1. Chemicals and Extenders

Routine chemicals and reagents were purchased from Sigma-Aldrich^®^ Agricultural Technology Development Co., Ltd. (Shanghai, China), Vazyme Biotechnology Development Co., Ltd. (Nanjing, China), and Nanjing Jiancheng Bioengineering Institute Co., (Nanjing, China). Pyrroloquinoline quinone disodium salt (PQQ) was purchased from MedChemExpress Co., Inc. (Monmouth Junction, NJ, USA).

### 2.2. Semen Collection and Processing

Semen was collected from ten healthy and fertile rams (small-tailed Han sheep) aged about 2 years twice a week using an artificial vagina during the breeding season (November 2022) at the Hongde livestock farm (Shouguang, China). A total of 80 ejaculates were used in this study. Rams were raised separately under natural light and fed with a commercial diet with unrestricted access to water. Semen collection from each ram was meticulously executed using an artificial vagina. The fresh semen was evaluated after collection, and only samples with motility over 90% were used in this study. The ejaculates were pooled to minimize individual variations and divided into 5 parts. Each part of semen was diluted with TCG solution containing various concentrations of PQQ (0, 10, 100, 1000, 10,000 nM) at a concentration of 100 × 10^6^ sperm/mL. The TCG solution was composed of 250 mM Tris, 83 mM citric acid, and 69 mM D-glucose. Diluted semen was stored at 4 °C in a cool incubator (sc-320D, Haier, Qingdao, China) for 96 h before evaluation.

### 2.3. Evaluation of Sperm Motility by Computer-Assisted Sperm Analysis (CASA) System

CASA was used to assess sperm motility as described in a previous study [34]. Aliquots of 5 μL sperm samples were placed in the pre-warmed analyzer’s Makler chamber (10 µm depth; Haifa Instruments, Haifa, Israel). Evaluations were made at 10× magnification. Thereafter, three regions were randomly selected for analysis. More than 500 sperm cells were evaluated. Recorded parameters were curvilinear velocity (VCL, μm/s), straight-line velocity (VSL, μm/s), average path velocity (VAP, μm/s), linearity (LIN, %), straightness (STR, %), lateral head displacement (ALH, μm), beat cell frequency (BCF, Hz), progressive motility (%), and total motility (%). VCL, VSL, STR, LIN, VAP, ALH, and BCF were only calculated for motile sperm.

### 2.4. Detection of Sperm Plasma Membrane Integrity and Acrosome Integrity

As described in previous studies [35,36], sperm membrane integrity and acrosome integrity were detected with a live/dead sperm motility assay kit (L-7011, Thermo Fisher, Shanghai, China) and fluorescein isothiocyanate-peanut lectin (L-7381, Sigma-Aldrich, Shanghai, China), respectively. For membrane integrity evaluation, sperm samples were incubated with 100 nM SYBR-14 working solution and 2.4 mM PI solution in the dark for 10 min. For acrosome integrity detection, sperm samples were incubated with fluorescein isothiocyanate-peanut lectin solution (100 µg/mL). A fluorescence microscope at 400× magnification was used to evaluate acrosome integrity and plasma membrane integrity (emitting green fluorescence at 516 nm and red fluorescence at 617 nm). More than 500 sperm cells were evaluated.

### 2.5. Evaluation of Mitochondrial Membrane Potential

The JC-1 mitochondrial membrane potential detection kit (Beyotime Institute of Biotechnology, Shanghai, China) was used to analyze sperm mitochondrial membrane potential (ΔΨm) as described in our previous study [37]. Sperm samples were incubated with JC-1 working solution following the manufacturer’s instructions. The monomer and aggregates of the two types of JC-1 in stained mitochondrial plasma emit green fluorescence in low ΔΨm and emit red fluorescence in high ΔΨm, respectively. More than 500 sperm cells were evaluated.

### 2.6. Detection of Sperm Reactive Oxygen Species

A reactive oxygen species (ROS) assay kit (S0033S, Beyotime, Shanghai, China) was used to analyze sperm ROS levels following the manufacturer’s instructions. Sperm samples were incubated with 200 μL of DCFH-DA reagent working solution at 37 °C for 10 min in the dark. After that, the mitochondrial ROS level was analyzed by flow cytometry using a filter with a bandwidth of 525/26 nm and was measured as the mean fluorescence intensity (MFI). A total of 20,000 sperm-specific events were analyzed.

### 2.7. Measure of the Sperm ATP Level

An ATP assay kit (Beyotime Institute of Biotechnology) was used to measure sperm ATP levels as described in a previous study [38]. After lysis and centrifugation, 50 μL of the sperm supernatant was mixed with 100 μL luciferin/luciferase reagent in 96-well plates. An Ascent Luminoskan luminometer (Thermo Scientific, Palm Beach, FL, USA) was used to read the luminescence at integration × 1000 ms. Analyses were performed in triplicate (*n* = 3).

### 2.8. Measurement of Sperm MDA Levels

A malondialdehyde (MDA) assay kit (Nanjing Jiancheng Institute of Biotechnology, Nanjing, China) was used to measure sperm MDA levels as described in another study [39]. Sperm samples were lysed on ice. Each sample was mixed with reaction buffer reagent and boiled for 40 min. After cooling, the sample was centrifuged, and the supernatant was collected. Furthermore, absorbance was taken at 532 nm using a microplate reader (TECAN, Infinite M Nano, Männedorf, Switzerland). The analyses were performed in triplicate (*n* = 3).

### 2.9. Determination of SOD Activity

The sperm samples were homogenized in 100 mM Tris-HCl buffer and centrifuged at 1600× *g* for 10 min to collect the supernatant, then the SOD activity was measured using a detection kit (Jiancheng Institute of Bioengineering, Nanjing, China) as described by Zhang et al. (2022) [40]. The absorbance was taken with a microplate reader at 450 nm (TECAN, Infinite M Nano, Männedorf, Switzerland). Analyses were performed in triplicate (*n* = 3).

### 2.10. Western Blotting

Total sperm protein extraction was carried out in a sodium dodecyl sulfate (SDS) sample buffer. Samples were solubilized in lysis buffer containing 1% protease inhibitor cocktail (Sigma) in ice for 30 min, followed by sonication for 1 min which was carried out twice at an interval of 5 min. The supernatants were collected after centrifugation at 10,000× *g* for 15 min at 4 °C. The proteins were separated by 5% SDS-PAGE and transferred to nitrocellulose (PVDF) membranes (GE Bioscience, Newark, NJ, USA). Non-specific binding sites were blocked by incubation in Tris-buffered saline (TBS) containing 0.1% (*v*/*v*) Tween-20 and 5% (*w*/*v*) bovine serum albumin (Life Technologies, Grand Island, NY, USA). The membranes were immunoblotted with primary antibodies anti-mitochondrial NADPH dehydrogenase subunits 1 (anti-MT-ND1; WH225135, Aibotech, Shenzhen, China), anti-mitochondrial NADPH dehydrogenase subunits 6 (anti-MT-ND6; bs-22462, Aibotech, China), anti-phosphotyrosine (EPR16871, Abcam, Waltham, MA, USA), and anti-α-tubulin (MA5-16308; Thermo Fisher, Waltham, MA, USA) diluted in 5% bovine serum albumin in TBS-Tween (1:1000 dilution) overnight at 4 °C, followed by incubation with the HRP-conjugated secondary antibody (goat anti-rabbit antibody for MT-ND1, MT-ND6, anti-phosphotyrosine, α-tubulin, 1:1000 dilution). After washing in TBST, enhanced chemiluminescence (ECL) detection was performed using the ECL system following the manufacturer’s specifications (GE Bioscience), and appropriate exposure of blots to Fuji X-ray film (Fujifilm, Tokyo, Japan). Band intensities were analyzed using a Gel-Pro analyzer (Media Cybernetics, Rockville, MD, USA).

### 2.11. Immunoprecipitation

Total sperm protein was extracted from goat semen samples in a cell lysis buffer. A total of 100 μL sperm lysate was incubated overnight with 4-hydroxynonenal (4-HNE, MA5-27570, Thermo Fisher) (1:25) at 4 °C. Mouse IgG Ab was added (Magnetic Bead Conjugate) (5873 s, Cell Signaling Technology; add 1:20 Paint thinner), and shaking was carried out gently at 4 °C for 3 h. Thereafter, the sample was centrifuged (10,000× *g*, 4 °C, 10 min) and the supernatant was discarded for precipitation. Then, the sample was resuspended using 20 μL SDS (1×) sample buffer for western blotting analysis of immunoprecipitation (IP) results.

### 2.12. Sperm Capacitation

The TALP medium contained 100 mM NaCl, 3.1 mM KCl, 25 mM NaHCO_3_, 0.3 mM NaH_2_PO_4_, 21.6 mM Na lactate, 3 mM CaCl_2_, 0.4 mM MgCl_2_, 10 mM HEPES, 1 mM Na pyruvate, 5 mM glucose, and 5 mg/mL bovine serum albumin (BSA), with a pH of 7.3. Sperm samples were incubated with TALP medium at 39 °C in a humidified incubator with 5% CO_2_ in the air for 3 h. 1 mM caffeine, 1 mM theophylline, and 2.5 mM methyl-b-cyclodextrin were added to the TALP medium to induce ram sperm capacitation in vitro as described in a previous study [41].

### 2.13. Chlortetracycline (CTC) Analysis

As described in a previous study [36], the CTC staining solution contained 20 mM Tris, 130 mM NaCl, 5 mM cysteine, and 750 μM CTC. The fixed solution contained 1% (*v*/*v*) glutaraldehyde in a 1 M Tris-HCl buffer (pH 7.4). For staining, 50 μL of sperm suspension was mixed with 100 μL CTC staining solution. Then, the sperm sample was evaluated with a fluorescence microscope at 400× magnification. At least 500 sperm cells were evaluated. 

### 2.14. Sperm–Zona Pellucida (ZP) Binding Capacity

The porcine ovaries were collected from an abattoir and transported to the laboratory in phosphate-buffered saline (PBS) at 36–38 °C. Cumulus–oocyte complexes were obtained by aspirating 3–8 mm follicles. As described by previous studies [42,43,44,45,46], the cumulus–oocyte complexes were isolated and incubated in PBS (Solarbio, Beijing, China) supplemented with 3 mg/mL hyaluronidase at 38.5 °C for 5 min, followed by gentle pipetting for 3 to 5 min to remove cumulus cells. The zona pellucidas were prepared from the cumulus-cell-free oocytes using a microinjector (Biocompare, San Francisco, CA, USA). Twenty zona pellucidas were placed in a 1000 μL droplet of culture medium for each group. Meanwhile, the sperm samples were incubated with capacitation medium. Then, 50 μL of the capacitated sperm suspension was added into the PBS droplet containing 20 zona pellucidas, and incubated for 6 h at 38.5 °C in a humidified atmosphere saturated with 5% CO_2_. Following incubation, the sperm–zona pellucida complexes were gently rinsed three times with PBS using a bore pipette to remove the loosely attached sperm. The sperm tightly bound to each ZP was also removed by repeated aspiration using a narrow-bore pipette, and were counted under a microscope. The total number of sperm bound to ZP were counted.

### 2.15. Statistical Analysis

Data from three replicates were compared using either Student’s *t*-test or one-way analysis of variance followed by Tukey’s post hoc test (Statview; Abacus Concepts, Inc., Berkeley, CA, USA). All values are presented as the mean ± standard deviation (SD). Differences between treatments were considered statistically significant at *p* < 0.05.

## 3. Results

### 3.1. PQQ Improved Sperm Motility Parameters during Storage at 4 °C

As shown in Table 1, compared to the control, the addition of 100 nM PQQ to the medium significantly (*p* < 0.05) increased the sperm total motility (TM), progressive motility (PM), curvilinear velocity (VCL), straight-line velocity (VSL), average path velocity (VAP), straightness (STR), and linearity (LIN) at 24 h, 48 h, 72 h, and 96 h points of storage; meanwhile, there was no significant difference (*p* > 0.05) in beat-cross frequency (BCF) and lateral head (ALH) parameters. Interestingly, the results of these parameters in 10 nM and 1000 nM PQQ treatments were not different from the control. Moreover, the addition of 10,000 nM PQQ significantly decreased (*p* < 0.05) ram sperm VCL, VSL, and VAP during the 96 h of preservation when compared to the control.

### 3.2. PQQ Improved Ram Sperm Membrane Integrity and Acrosome Integrity during Storage at 4 °C

**Figure 1:**
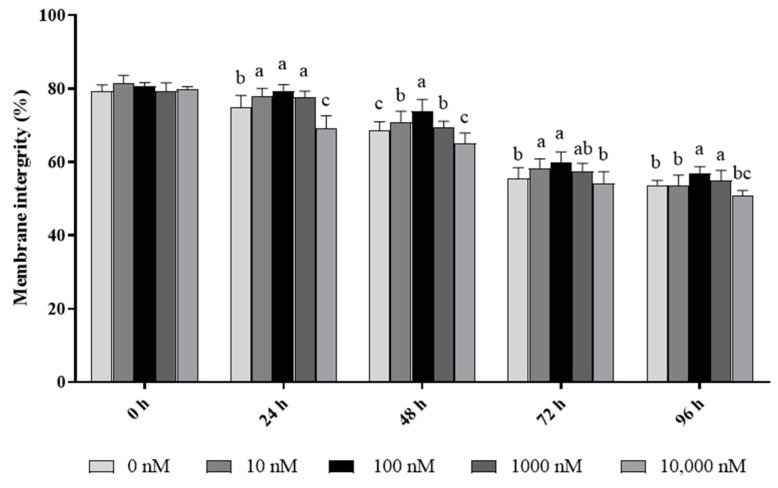
Effect of different concentrations of PQQ on ram sperm membrane integrity. Values are specified as mean ± standard deviation (SD). Columns with different lowercase letters differ significantly (*p* < 0.05), *n* = 5.

As shown in Figure 1, the ram sperm membrane integrity significantly (*p* < 0.05) decreased during 96 h of storage at 4 °C. The addition of PQQ ranging from 10 nM to 1000 nM doses significantly (*p* < 0.05) increased the value of sperm with membrane integrity during the 96 h period of storage 4 °C when compared to the control. Interestingly, the values of the percentage of sperm with membrane integrity in the 100 nM treatment were the highest among all the treatments. However, the addition of 10,000 nM PQQ treatment decreased (*p* < 0.05) the sperm membrane integrity at 24 h and 48 h points of storage, but there was no difference between the control and the groups treated with PQQ at 72 h and 96 h points of storage.

**Figure 2:**
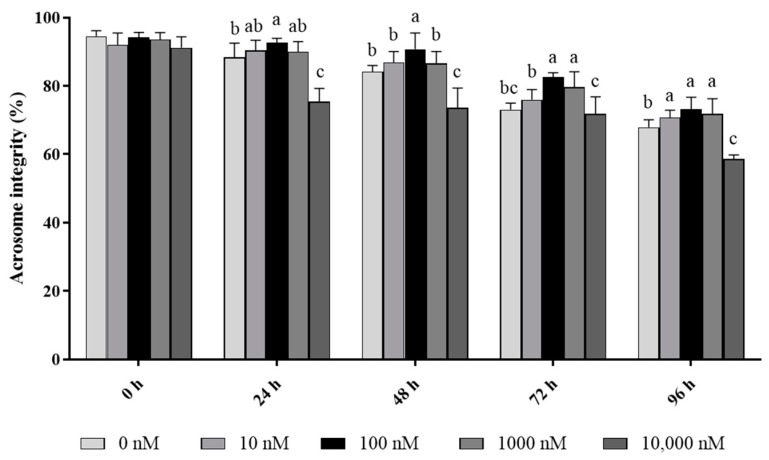
Effect of different concentrations of PQQ on ram sperm acrosome integrity. Values are specified as mean ± standard deviation (SD). Columns with different lowercase letters differ significantly (*p* < 0.05), *n* = 5.

In terms of acrosome integrity, it was observed that the addition of 10 to 1000 nM PQQ significantly increased (*p* < 0.05) the ratio of ram sperm with intact acrosomes during 96 h of storage, while the 10,000 nM PQQ treatment decreased the percentage of acrosome integrity compared to the control (Figure 2). In addition, 100 and 1000 nM PQQ treatments significantly increased (*p* < 0.05) acrosome integrity at 72 h point of storage while 10 and 1000 nM PQQ were not different from the control. In the 24 h and 48 h points of storage, 100 nM PQQ had the highest percentage for acrosome integrity while the other doses were not different from the control except for 10,000 nM PQQ which had a lower percentage (Figure 2).

### 3.3. PQQ Improved ram Sperm Mitochondrial Membrane Potentials and ATP Levels during Storage at 4 °C

**Figure 3:**
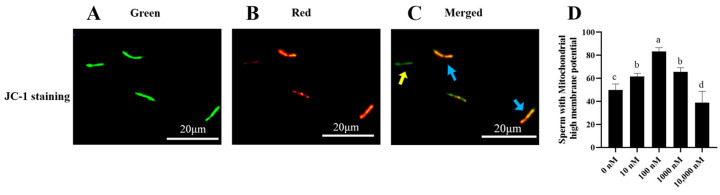
Effect of different concentrations of PQQ on ram sperm mitochondrial membrane potential (MMP). (**A**–**D**) Fluorescence microscopic observation of the mitochondrial membrane potential of ram sperm. (**A**): The monomer emits green fluorescence; (**B**): The aggregates emit red fluorescence; (**C**): Blue arrows indicate sperm with high MMP, meanwhile the yellow arrow indicates sperm with low MMP; (**D**): The values of different concentrations of PQQ on ram sperm mitochondrial membrane potential. Values are specified as mean ± standard deviation (SD). Columns with different lowercase letters differ significantly (*p* < 0.05), bars = 20 μm, *n* = 5.

As shown in Figure 3A–C, sperm that emits green fluorescence indicates sperm with low mitochondrial membrane potential, while sperm that emits red or orange fluorescence indicates sperm with high mitochondrial membrane potential. It was observed that the addition of 10 nM, 100 nM, and 1000 nM PQQ to the diluted medium significantly (*p* < 0.05) increased ram sperm mitochondrial membrane potentials compared to that of the control (Figure 3D). The 100 nM PQQ treatment presented the highest value of ram sperm with high mitochondrial membrane potential among all the treatments (Figure 3D). This shows that PQQ treatments increased the mitochondrial membrane potential, ATP levels, and reduced mitochondrial ROS levels. The damage of ROS to mitochondrial activity was alleviated; however, the 10,000 nM PQQ treatment was not different from the control in terms of sperm mitochondrial membrane potentials (Figure 3D).

**Figure 4:**
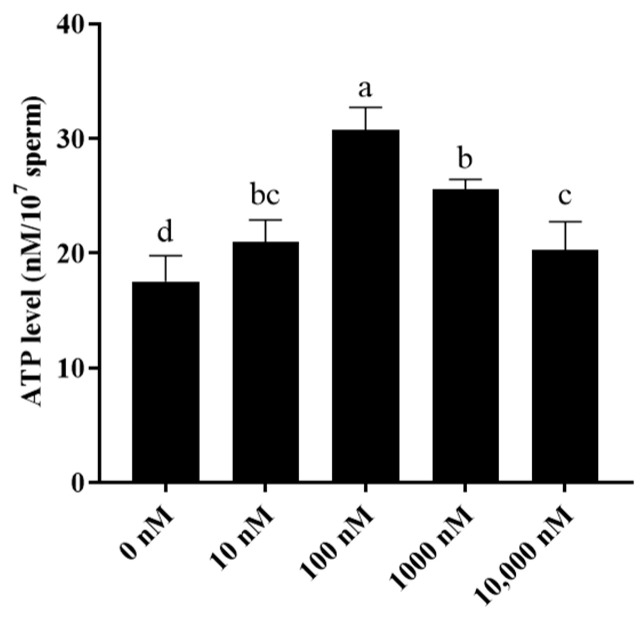
Effect of PQQ on ram sperm ATP level after storage at 4 °C. Values are specified as mean ± standard deviation (SD). Columns with different lowercase letters differ significantly (*p* < 0.05), *n* = 3.

Additionally, as mitochondria are important for sperm ATP generation, sperm ATP levels were also evaluated. It was observed that the addition of PQQ to the medium significantly increased sperm ATP levels when compared to that of the control with the 100 nM PQQ treatment having the highest level (Figure 4).

### 3.4. PQQ Reduced the Ram Sperm Oxidative Stress during Storage at 4 °C

**Figure 5:**
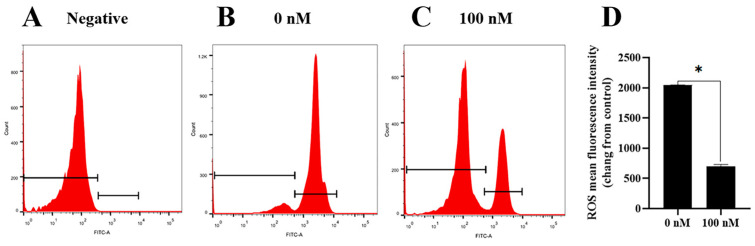
Effect of PQQ on ram sperm ROS level. (**A**): negative control; **(B**): Sperm treated without PQQ; (**C**): sperm treated with 100 nM PQQ; (**D**): The values of PQQ on ROS level. Values are specified as mean ± standard deviation (SD). Values are specified as mean ± standard deviation (SD). * differ significantly (*p <* 0.05).ROS: reactive oxygen species.

**Figure 6:**
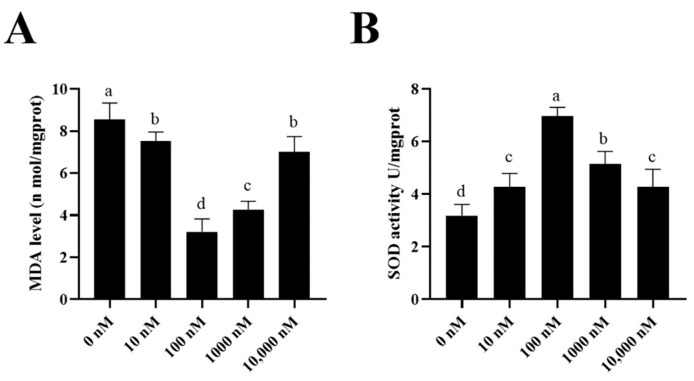
Effect of supplementation of PQQ sperm MDA levels (**A**) and SOD activity (**B**). Values are specified as mean ± standard deviation (SD). Columns with different lowercase letters differ significantly (*p* < 0.05). MDA: malondialdehyde; SOD: superoxide dismutase; *n* = 3.

To investigate how the PQQ improved the ram sperm quality, ram sperm mitochondrial ROS was measured. As shown in (Figure 5A–C), compared to the control, the addition of 100 nM PQQ to the medium significantly decreased the ROS levels. Also, the MDA levels in PQQ treatments were lower than those without PQQ. In addition, the values of SOD activity in the PQQ treatments were higher than those without PQQ (Figure 6B). Among the PQQ treatments, the 100 nM PQQ treatment had the lowest and highest MDA and SOD levels, respectively (Figure 6A).

### 3.5. PQQ Maintained Ram Sperm Mitochondrial Protein Levels by Decreasing ROS-Induced Protein Damage

**Figure 7:**
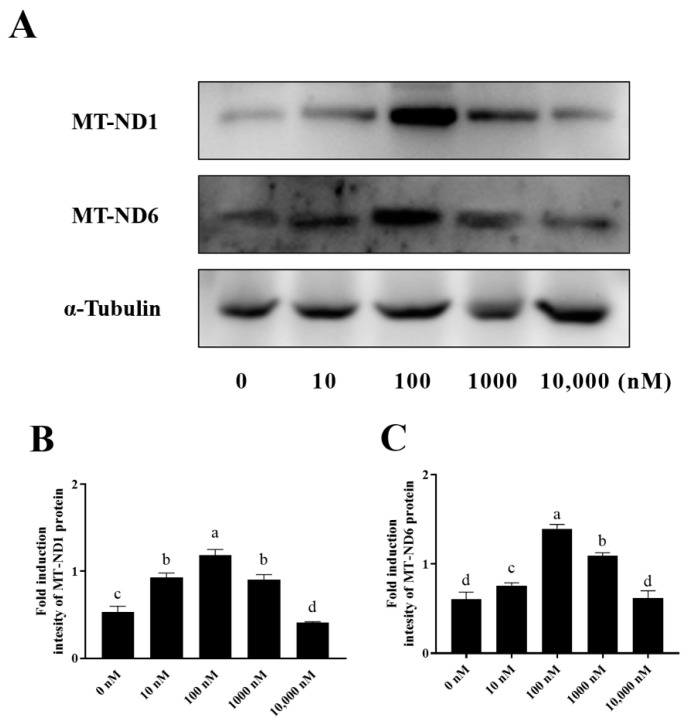
Effect of PQQ on ram sperm MT-ND1 and MT-ND6 protein expression. (**A**): Western blotting analysis of MT-ND1 and MT-ND6 in goat sperm mitochondrial; (**B**): Image J 1.8.0 analysis showing the grey value of MT-ND1; (**C**): Image J analysis showing the grey value of MT-ND6.Values are specified as mean ± standard deviation (SD). Columns with different lowercase letters differ significantly (*p* < 0.05), *n* = 3. MT-ND1: NADH dehydrogenase 1; MT-ND6: NADH dehydrogenase 6.

NADH dehydrogenase 1 (MT-ND1) and NADH dehydrogenase 6 (MT-ND6), which are mitochondrial proteins involved in the electron transfer through the respiratory chain, were also detected in this study. As shown in Figure 7A–C and Supplementary Figure S1, it was observed that the addition of 10 nM, 100 nM, and 1000 nM PQQ significantly (*p* < 0.05) increased the levels of MT-ND1 and MT-ND6 proteins compared to the control (0 nM), however, the 10,000 nM PQQ treatment did not lead to any increase in MT-ND1 and MT-ND6.

**Figure 8:**
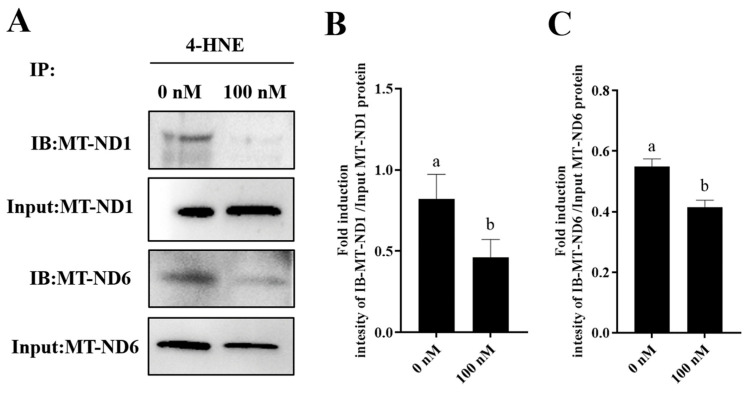
Effect of PQQ on ROS-induced damage of MT-ND1 and MT-ND6 proteins. (**A**):The immunoprecipitates were immunoblotted (IB) with antibodies specific for the indicated proteins (MT-ND-1, MT-ND6); (**B**): Image J analysis showing the grey value of MT-ND1; (**C**): Image J analysis showing the grey value of MT-ND6; Values are specified as mean ± standard deviation (SD). Columns with different lowercase letters differ significantly (*p* < 0.05). IB: immunoblotted; 4-HNE: 4-hydroxynonenal.

Furthermore, to elucidate the mechanism by which PQQ maintained the sperm MT-ND1 and MT-ND6 protein levels, we detected the damage of MT-ND1 and MT-ND6 proteins by IP. IP was performed using 4-HNE antibody and analyzed by WB with MT-ND1 and MT-ND6 antibodies. 4-HNE is considered a biomarker for lipid peroxidation when the cell suffers oxidative stress. And the 4-HNE is highly reactive and forms adducts with cellular proteins that lead to protein damage. As showed in Figure 8A–C and Supplementary Figure S2, the addition of 100 nM PQQ significantly decreased the ROS-induced damage of MT-ND1 and MT-ND6 proteins during storage, suggesting that the PQQ helped to reduce sperm ROS levels, and then reduced the 4-HNE generation which resulted in decreasing the binding of MT-ND1 and MT-ND6 proteins to 4-HNE.

### 3.6. The Addition of PQQ Increased Ram Sperm with Capacitation after Inducing Capacitation In Vitro

**Figure 9:**
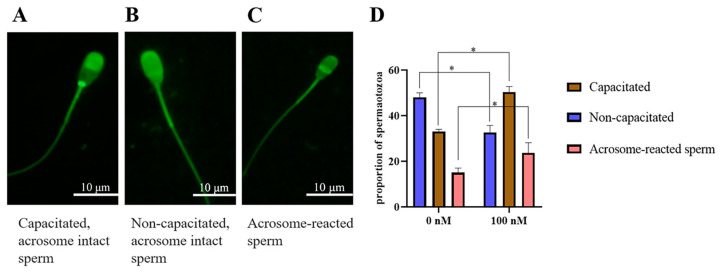
Three patterns of CTC fluorescence staining observed on ram sperm. (**A**): Capacitated, acrosome-intact sperm, with a fluorescence-free band in the post acrosomal region; (**B**): Non-capacitated, acrosome-intact sperm, with fluorescence over the whole head; (**C**): Acrosome-reacted sperm, with dull fluorescence over the whole head except for the thin, bright punctate band of fluorescence in the equatorial segment. Effect of PQQ on ram sperm capacitation (**D**). * differ significantly (*p* < 0.05), bars = 10 μm.

**Figure 10:**
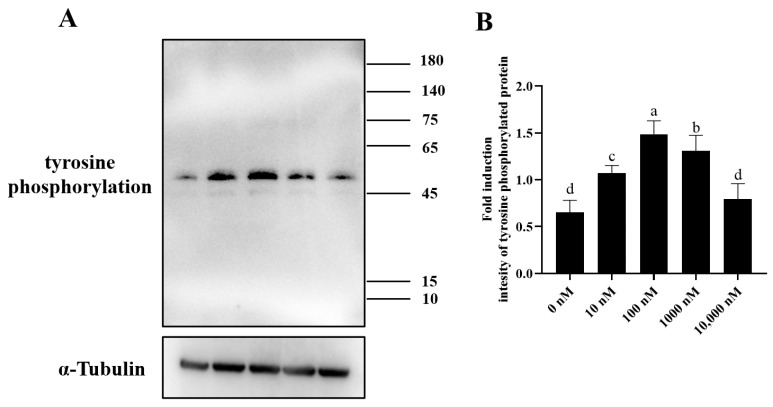
Effect of PQQ on ram sperm tyrosine phosphorylation protein expression after the sperm induced capacitation. (**A**): Western blotting analysis of tyrosine phosphorylation protein in ram sperm; (**B**): Image J analysis showing the grey value of tyrosine phosphorylation protein; Values are specified as mean ± standard deviation (SD). Columns with different lowercase letters differ significantly (*p* < 0.05), *n* = 3.

Chlortetracycline (CTC) is considered the gold standard in fluorescent microscopy analysis of sperm capacitation states. As showed in Figure 9A–C, there were three patterns of sperm observed after CTC staining: capacitated sperm, non-capacitated sperm, and acrosome-reacted sperm. After the ram sperm induced capacitation in vitro, the addition of 100 nM PQQ significantly (*p* < 0.05) increased the percentage of capacitated sperm and acrosome-reacted sperm when compared to that in the non-PQQ treatment (Figure 9D). Meanwhile, the percentage of non-capacitated sperm in PQQ treatment was lower than that in the non-PQQ treatment. In addition, as shown in Figure 10A,B and Figure S3, after each sperm sample induced capacitation, it was observed that the addition of PQQ (ranging from 10 nM to 1000 nM) significantly (*p* < 0.05) increased the level of tyrosine phosphorylated protein when compared to the control.

### 3.7. PQQ Improved Sperm–Zona Pellucida Binding Capacity

**Figure 11:**
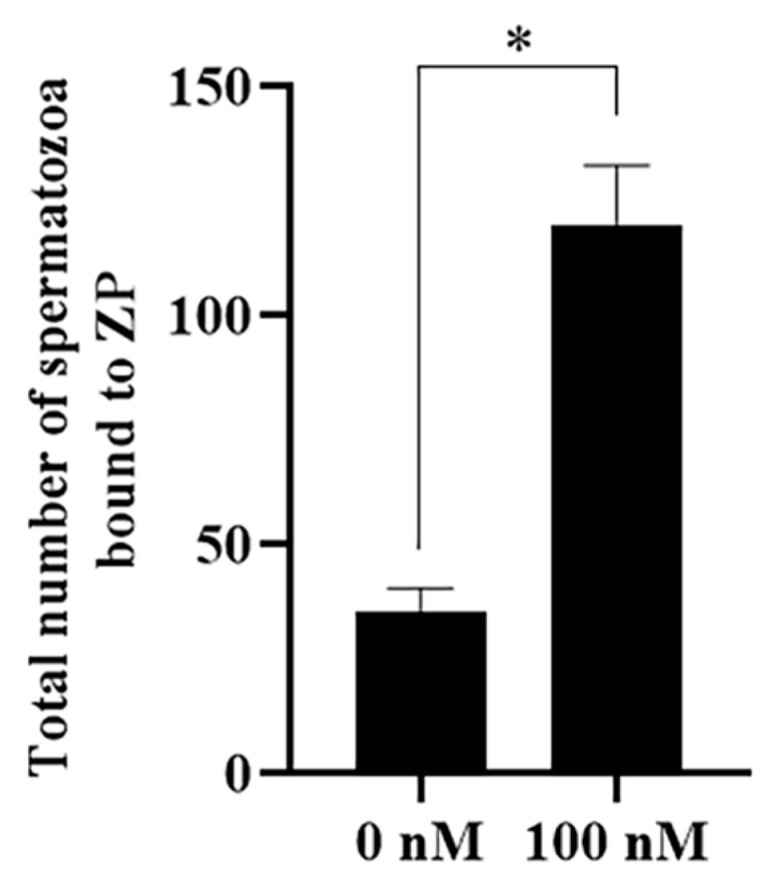
Effect of PQQ on sperm–zona pellucida (ZP) binding capacity after sperm was treated with 100 nM PQQ. * differ significantly (*p* < 0.05).Bars represent the mean ± standard deviation (SD) (*n* = 3, each replicate was performed on 20 zona pellucidas).

The effect of PQQ on sperm–zona pellucida (ZP) binding capacity was detected to evaluate sperm fertility after storage. As shown in Figure 11 and Figure S4, the addition of PQQ significantly increased the number of sperm bound to ZP (121.2 ± 2.7 vs. 30.6 ± 3.0, *p* < 0.05).

## 4. Discussion

Mitochondria, membrane-bound organelles in eukaryotic organisms, are regarded as powerhouses due to their central role in energy production [47]. Mitochondria are not only involved in ATP generation but they are also associated with various cellular functions, such as cellular calcium homeostasis [48], lipid homeostasis [49], generation of ROS [50], steroid hormone biosynthesis [51], and intrinsic apoptotic pathway [7,52]. Ensuring the proper function of the mitochondria is important in maintaining cell survival. Previous studies reported that mitochondrial dysfunction disrupts the activity of cells, tissues, and organs, and underlies a remarkably wide range of pathologies [53]. In sperm, mitochondrial function has been associated with sperm quality and fertilization ability [54]. Mundy et al. (1995) and Pelliccione et al. (2011) found that the defects in mitochondrial ultrastructure decrease sperm motility [55,56]. In this study, it was observed that in the control group, sperm mitochondrial membrane potential and motility decreased. Generally, sperm needs ATP to support its movement. ATP is generated from sperm glycolysis and oxidative phosphorylation (OXPHOS) pathways. The ATP generated from the mitochondria is the main energy source for supporting sperm motility. Our previous study showed the relationship between both parameters; when boar sperm was treated with a low glucose medium to activate mitochondrial activity, sperm generated more ATP [33]. Similarly, the results in this study showed a relationship between ATP levels and mitochondrial membrane potential, as both parameters increased with PQQ treatment. This suggests that PQQ supplementation improves ram sperm mitochondrial membrane potential to generate increased amounts of ATP, culminating in increased sperm motility.

NADH dehydrogenase (Complex I), succinate dehydrogenase (Complex II), cytochrome bc1 (Complex III), and cytochrome oxidase (Complex IV) are the respiratory enzyme complexes of the electron transport chain (ETC) in the inner mitochondrial membrane [57]. The mitochondrial membrane potential relies on ETC subunit status [58], and MT-ND1 and MT-ND6 proteins are the subunits of the ETC. Zhu et al. reported that boar sperm mitochondrial membrane potential was reduced when ETC subunits were damaged [33]. The present study agrees with Zhu et al. as the levels of MT-ND1 and MT-ND6 proteins in PQQ treatment were higher than those of the control. Notably, sperm mitochondrial ETC subunits may regulate mitochondrial function, thereby influencing the quality of sperm. Amaral et al. reported that the expression of mitochondrial proteins, and notably ETC subunits, were associated with sperm quality [59]. Previous comparative proteomic outcomes revealed that the expression of several sperm mitochondrial proteins in asthenozoospermic patients was different from normal sperm [60,61,62,63,64]. Ruiz-Pesini et al. (1998) demonstrated that the reduction of sperm mitochondrial enzyme activity, including ETC complexes, decreased sperm vitality and motility [65]. In this study, PQQ improved the expression of ram sperm MT-ND1 and MT-ND6 proteins, hence maintaining the sperm’s high mitochondrial membrane potential. Furthermore, it was observed that PQQ treatment decreased the oxidative damage of MT-ND1 and MT-ND6 proteins. By preventing the damage of mitochondrial MT-ND1 and MT-ND6 proteins, PQQ maintains ram sperm quality during storage at 4 °C.

ROS generation and accumulation are expected during sperm storage in vitro. Previous studies reported that excessive ROS can damage sperm proteins [66], lipids [67], and DNA [68], leading to decreased sperm motility [69]. The sperm membrane is sensitive to ROS, and unsaturated fatty acids on the membrane are easily attacked by ROS, causing the sperm membrane to lose its fluidity, thereby decreasing membrane and acrosome integrity [69,70]. In this present study, the control had a higher ROS level compared to the PQQ treatment, whereas values for both membrane integrity and acrosome integrity for the PQQ treatment were higher than the control. Therefore, PQQ supplementation can increase ram sperm membrane integrity and acrosome integrity by reducing ROS damage during storage in vitro.

Sperm capacitation is an important physiological prerequisite before acrosomal reactions and egg fertilization [71]. Only capacitated sperm has the exclusive ability to undergo acrosome reaction and subsequently fertilize the egg. In the present study, after each treatment sperm sample induced capacitation in vitro, it was found that the 100 nM PQQ treatment significantly increased the percentage of capacitated sperm and acrosome-reacted sperm when compare to that of the control. And the value of sperm tyrosine phosphorylation proteins in the 100 nM PQQ treatment was also higher than that of the control. These data indicated that the addition of 100 nM PQQ to the diluted medium maintained ram sperm fertility. Indeed, it was observed that the sperm–zona pellucida binding capacity in PQQ treatment was higher than in the non-PQQ treatment. Therefore, during ram sperm storage at 4 °C, the addition of PQQ might maintain sperm fertility by decreasing the damage induced by ROS. 

## 5. Conclusions

**Figure 12:**
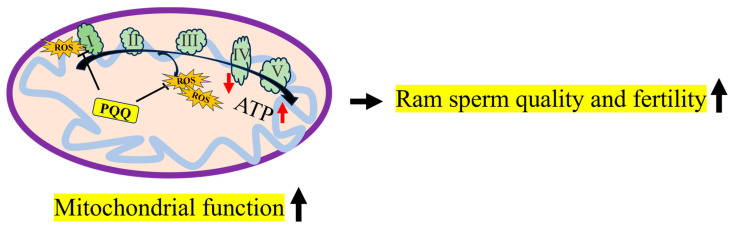
The mechanism of PQQ in maintaining ram sperm quality during storage at 4 °C. I: complex I; II: complex II; III: complex III; IV: complex IV; V: complex V; ROS: reactive oxygen species; PQQ: pyrroloquinoline quinone.

As shown in Figure 12, during ram sperm storage at 4 °C, sperm suffered oxidative stress, the excessive ROS levels damaged MT-ND1 and MT-ND6 proteins, considered to be the subunits of complex I that reduce the mitochondrial function. Moreover, the addition of PQQ protected the MT-ND1 and MT-ND6 proteins from damage induced by the ROS, and then preserved the high mitochondrial membrane potentials of the sperm for ATP generation, which resulted in maintaining sperm motility, membrane integrity, and fertility. PQQ supplementation is recommended during the storage of chilled ram sperm, and this will contribute to the improvement of sheep production.

## Figures and Tables

**Table 1:** Effect of PQQ on ram sperm motility parameters measured with CASA during storage.

Sperm Parameters	Time (h)	0 nM	10 nM	100 nM	1000 nM	10,000 nM
Total motility (%)	24	87.9 ±11.4 ^b^	90.0 ± 3.9 ^ab^	93.3 ± 1.2 ^a^	88.5 ± 3.0 ^b^	77.8 ± 4.3 ^c^
	48	74.9 ± 5.0 ^bc^	80.2 ± 6.1 ^b^	86.0 ± 7.4 ^a^	82.0 ± 6.5 ^b^	70.8 ± 3.4 ^c^
	72	59.2 ± 3.1 ^c^	67.3 ± 5.4 ^b^	72.1 ± 2.1 ^a^	71.4 ± 0.5 ^a^	58.3 ± 0.5 ^c^
	96	40.3 ± 0.5 ^b^	41.8 ± 1.6 ^ab^	43.6 ± 0.5 ^a^	38.8 ± 2.2 ^b^	39.2 ± 2.0 ^b^
Progressive motility (%)	24	60.5 ± 7.8 ^bc^	71.4± 4.1 ^b^	73.2 ± 4.1 ^a^	68.1 ± 1.6 ^b^	51.9 ± 1.8 ^c^
	48	53.5 ± 6.1 ^bc^	53.4 ± 7.2 ^bc^	71.3 ± 8.4 ^a^	57.2 ± 7.6 ^b^	41.4 ± 2.9 ^c^
	72	41.2 ± 5.2 ^b^	43.6 ± 1.3 ^ab^	48.5 ± 0.6 ^a^	45.1 ± 0.2 ^a^	39.6 ± 1.6 ^b^
	96	22.3 ± 0.7 ^b^	22.6 ± 1.2 ^b^	24.5 ± 1.4 ^a^	24.9 ± 0.7 ^a^	20.6 ± 0.6 ^c^
VCL (μm/s)	24	67.8 ± 3.5 ^b^	60.8 ± 8.7 ^b^	76.6± 6.0 ^a^	66.2± 3.0 ^b^	54.2 ± 1.2 ^c^
	48	67.6 ± 4.3 ^b^	70.2 ± 4.3 ^b^	78.9 ± 10 ^a^	74.9± 11.2 ^a^	63.6 ± 1.2 ^c^
	72	59.3 ± 3.6 ^b^	62.6 ± 0.3 ^b^	67.5 ± 8.2 ^a^	66.2 ± 4.9 ^a^	52.3 ± 0.5 ^c^
	96	36.5 ± 1.2 ^b^	36.3 ± 0.5 ^b^	42.7 ± 0.4 ^a^	38.5 ± 1.5 ^b^	32.5 ± 8.0 ^c^
VSL (μm/s)	24	38.5 ± 7.8 ^b^	47.2 ± 5.0 ^ab^	58.0 ± 3.1 ^a^	41.4 ± 4.2 ^b^	30.3 ± 2.2 ^c^
	48	36.7 ± 5.0 ^b^	35.8 ± 4.9 ^b^	45.5 ± 6.5 ^a^	36.4 ± 7.5 ^b^	29.6 ± 0.6 ^c^
	72	36.5 ± 4.2 ^b^	34.8 ± 4.1 ^b^	44.9 ± 5.6 ^a^	38.3 ± 7.8 ^b^	26.3 ± 2.9 ^c^
	96	18.5 ± 0.6 ^b^	20.3 ± 2.3 ^b^	22.6 ± 4.2 ^a^	29.2 ± 2.3 ^a^	15.6 ± 0.9 ^c^
VAP (μm/s)	24	47.6 ± 4.8 ^b^	47.2 ± 5.0 ^b^	61.6 ± 2.1 ^a^	38.6 ± 3.9 ^c^	33.4 ± 4.1 ^d^
	48	48.8 ± 11.0 ^b^	46.5 ± 6.5 ^b^	59.0 ± 9.2 ^a^	47.7 ± 8.9 ^b^	36.3 ± 0.7 ^c^
	72	45.2 ± 12 ^b^	47.8 ± 5.6 ^b^	55.4 ± 11.4 ^a^	41.6 ± 8.6 ^c^	31.2 ± 0.9 ^d^
	96	39.6 ± 0.5 ^b^	41.3 ± 5.2 ^b^	46.2 ± 4.2 ^a^	45.3 ± 0.7 ^a^	32.9 ± 5.8 ^c^
BCF (Hz)	24	24.7 ± 1.2	24.5 ± 1.4	26.4 ± 0.9	24.9 ± 0.6	24.2 ± 1.2
	48	21.3 ± 3.2	22.1 ± 4.1	22.5 ± 3.2	21.9 ± 0.9	20.6 ± 1.3
	72	22.3 ± 3.1	31.5 ± 1.6	23.6 ± 3.6	19.6 ± 0.8	22.3 ± 0.5
	96	22.6 ± 2.4	22.9 ± 1.2	24.5 ± 3.2	21.3 ± 1.4	26.1 ± 0.8
ALH (μm)	24	8.7 ± 0.6	8.3 ± 0.3	8.7 ± 0.4	8.2 ± 0.3	8.4 ± 0.3
	48	6.9 ± 0.5	7.2 ± 1.2	7.3 ± 1.3	6.9 ± 2.3	7.3 ± 1.2
	72	7.5 ± 0.9	7.4 ± 1.2	7.3 ± 1.6	7.8 ± 1.9	7.4 ± 1.6
	96	7.4. ± 1.4	8.3 ± 0.9	8.5 ± 2.1	7.6 ± 0.6	7.2 ± 1.5
STR (%)	24	79.7 ± 1.7 ^b^	76.4 ± 1.3 ^b^	81.4 ± 1.4 ^a^	79.7 ± 3.1 ^b^	75.6 ± 0.9 ^c^
	48	66.8 ± 4.3 ^b^	67.5 ± 2.3 ^b^	72.1 ± 1.7 ^a^	55.6 ± 1.7 ^c^	51.5 ± 0.8 ^d^
	72	71.3 ± 2.6 ^b^	74.9 ± 2.5 ^ab^	75.3 ± 0.9 ^a^	55.6 ± 0.9 ^c^	71.3 ± 1.4 ^b^
	96	54.1 ± 1.2 ^c^	58.6 ± 2.1 ^bc^	71.3 ± 0.8 ^a^	64.2 ± 1.5 ^b^	63.8 ± 1.8 ^b^
LIN (%)	24	49.2 ± 1.5 ^b^	42.2 ± 6.3 ^b^	60.1 ± 1.5 ^a^	46.5 ± 4.4 ^b^	41.2 ± 0.7 ^b^
	48	47.4 ± 0.3 ^ab^	47.4 ± 6.3 ^ab^	58.3 ± 5.2 ^a^	47.8 ± 5.3 ^a^	46.5 ± 0.8 ^b^
	72	46.9 ± 7.2 ^b^	47.3 ± 6.2 ^b^	55.3 ± 4.6 ^a^	47.8 ± 12.0 ^b^	46.2 ± 11.4 ^b^
	96	32.1 ± 0.5 ^c^	34.2 ± 0.8 ^c^	44.5 ± 1.6 ^a^	41.2 ± 0.6 ^b^	43.2 ± 1.2 ^a^

(Values are expressed as mean ± standard deviation. Different letters within a column indicate significant difference (*p* < 0.05). VCL, curvilinear velocity; VSL, straight-line velocity; VAP, average path velocity; BCF, beat-cross frequency; ALH, lateral head; STR, straightness (VSL/VAP); LIN, linearity (VSL/VCL)).

## Data Availability

The data presented in this study are available in the article.

## Supplementary Materials

The following supporting information can be downloaded at: https://www.mdpi.com/article/10.3390/antiox13010104/s1, Figure S1. Different concentrations of PQQ on the expression of proteins (MT-ND1, MT-ND6) in ram sperm stored at 4 °C. Figure S2. PQQ decreased the ROS damage of mitochondrial proteins (MT-ND1 and MT-ND6). Figure S3. Different concentrations of PQQ on the expression of tyrosine phosphorylation proteins in ram sperm stored at 4 °C. Figure S4. Effect of PQQ on sperm-zona pellucida (ZP) binding capacity after sperm treated with PQQ. (A) zona pellucida. (B) Control group sperm-zona pellucida complex, (C) 100 nM PQQ group sperm-zona pellucida complexes, red arrow indicate the sperm tightly bound to zona pellucida. Bars = 100 μm.
