# Supplementary material for: Pyrroloquinoline Quinone Improves Ram Sperm Quality through Its Antioxidative Ability during Storage at 4 °C
Source: Antioxidants (Basel). 2024 Jan 15;13(1):104. doi: 10.3390/antiox13010104 (PMC10812569; doi:10.3390/antiox13010104)
Supplement: Supplementary file 1 [file antioxidants-13-00104-s001.zip › antioxidants-2784540-supplementary.pdf]

Supplementary Figure S1. Different concentrations of PQQ on the expression of proteins (MT-ND1、MT-ND6) in ram sperm stored at 4℃.

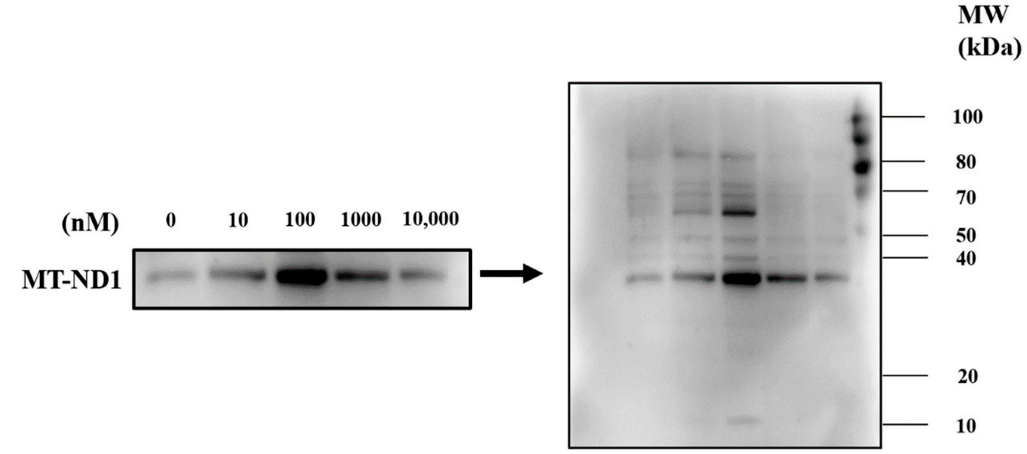

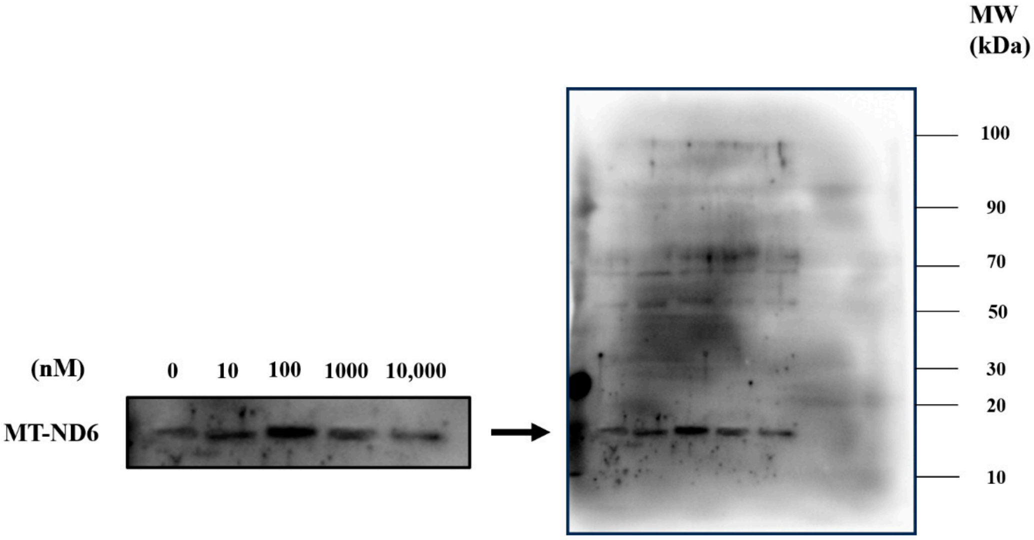

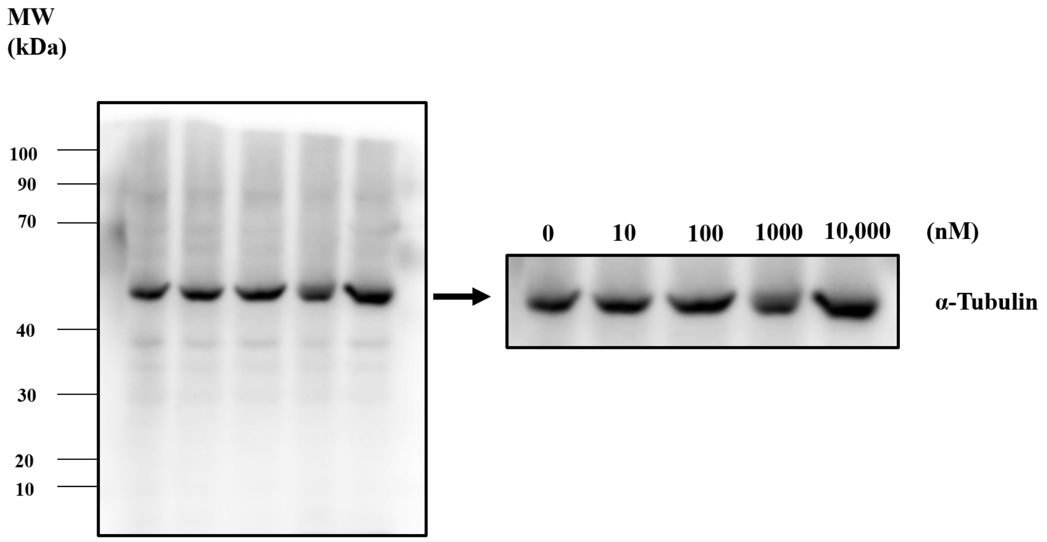
MW
(kDa)
100
90
70
40
30
20
10
0
10
100
1000
10,000
(nM)
α-Tubulin

Supplementary Figure S2. PQQ decreased the ROS damage of mitochondrial proteins (MT-ND1 and MT-ND6).

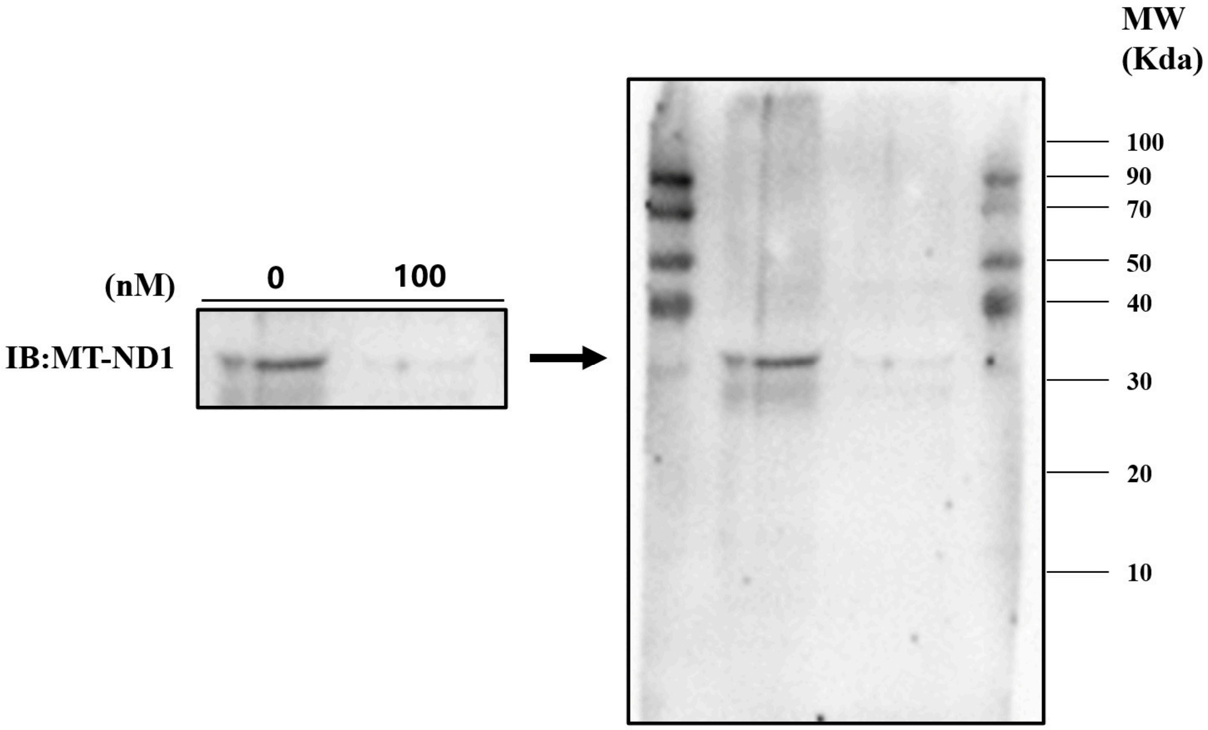

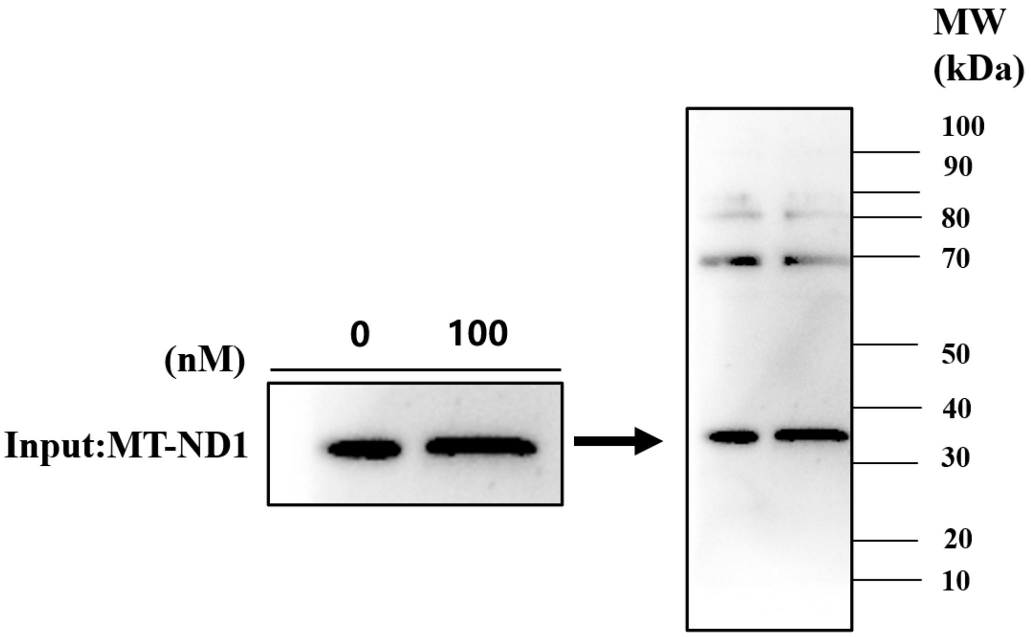

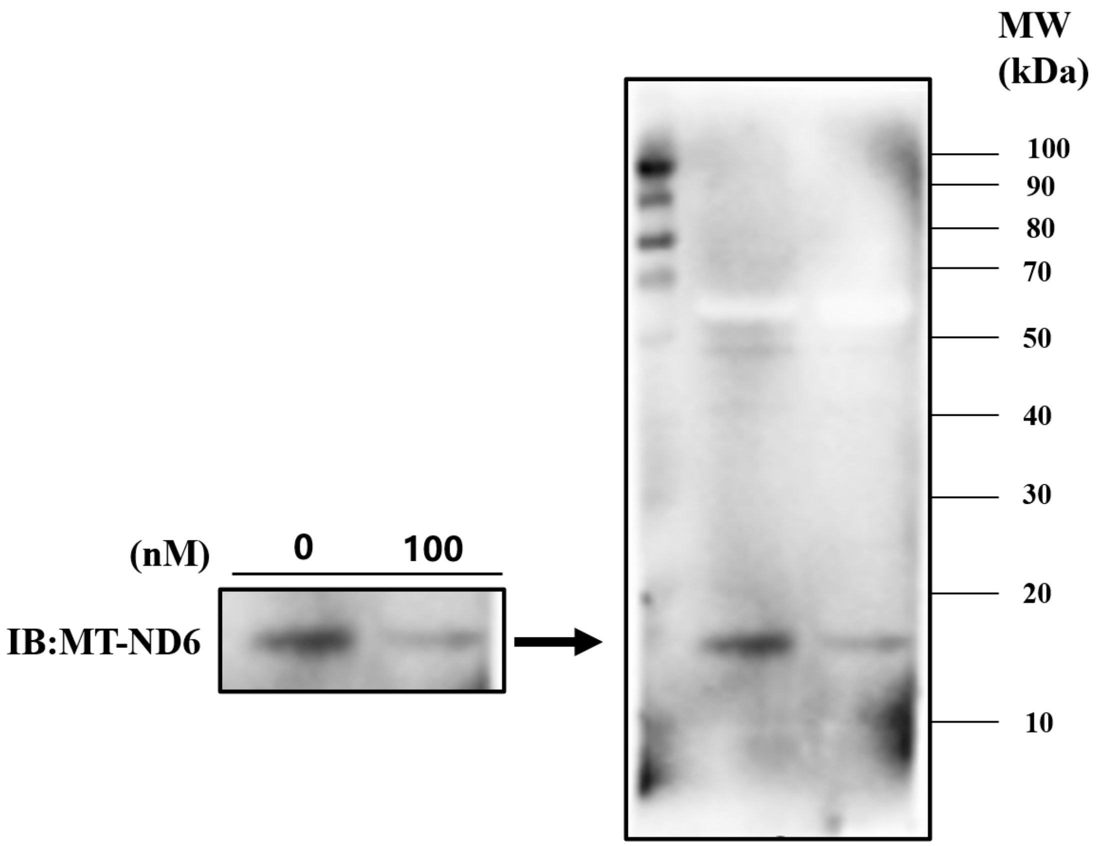
MW (kDa)
100
90
80
70
50
40
30
20
10
(nM)
0
100
IB:MT-ND6

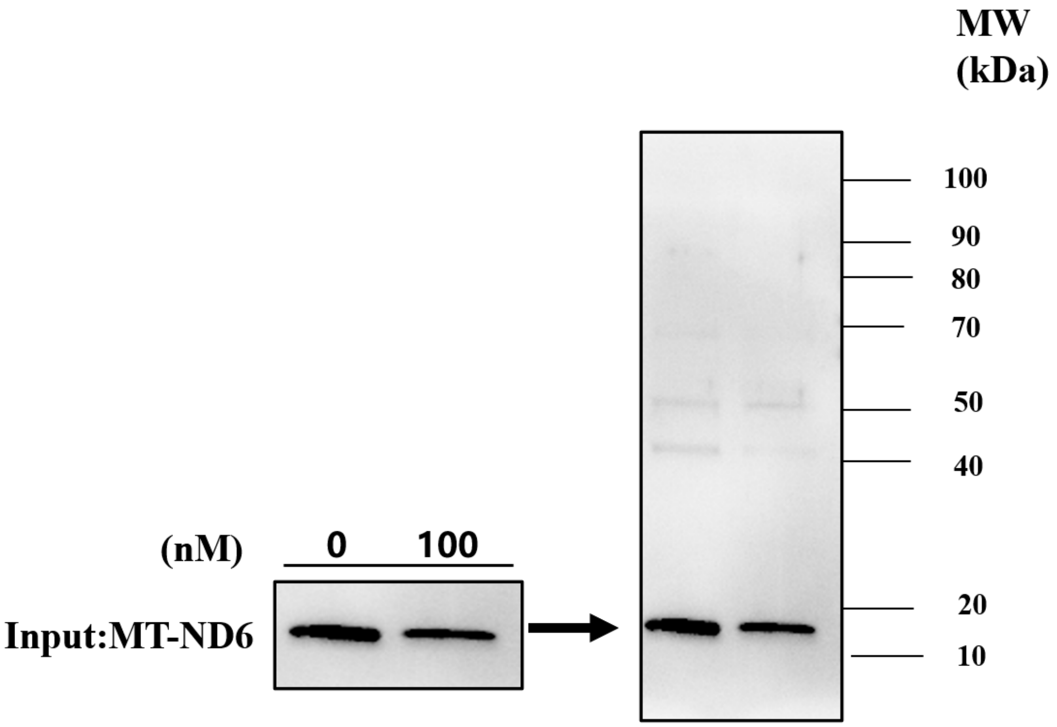
MW (kDa)
100
90
80
70
50
40
20
10
(nM)
0
100
Input:MT-ND6

Supplementary Figure S3. Different concentrations of PQQ on the expression of tyrosine phosphorylation proteins in ram sperm stored at 4℃

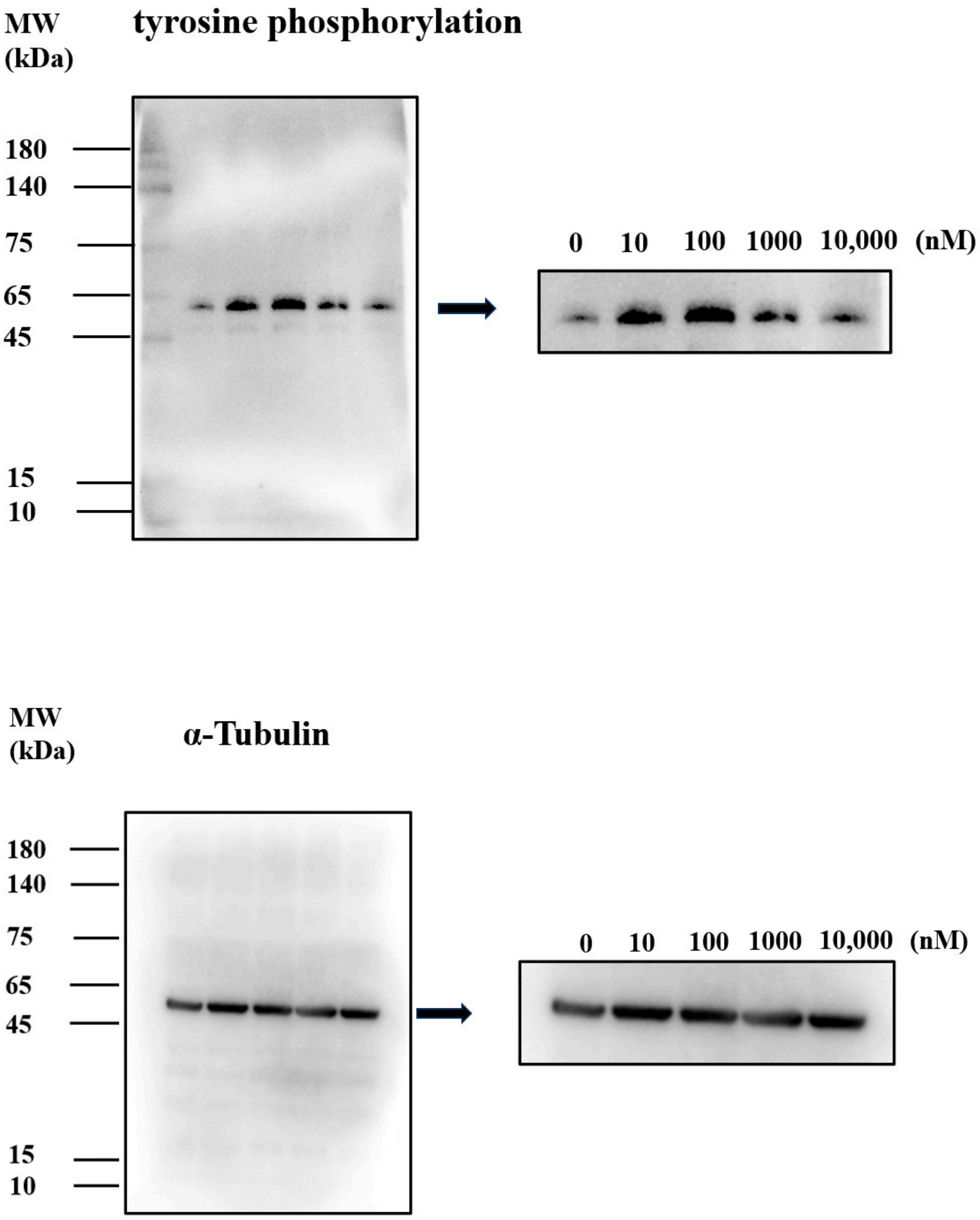

Supplementary Figure S4. Effect of PQQ on sperm-zona pellucida (ZP) binding capacity after sperm treated with PQQ. (A) zona pellucida. (B) Control group sperm-zona pellucida complex, (C) 100 nM PQQ group sperm-zona pellucida complexes, red arrow indicate the sperm tightly bound to zona pellucida. Bars=100 µm.

.

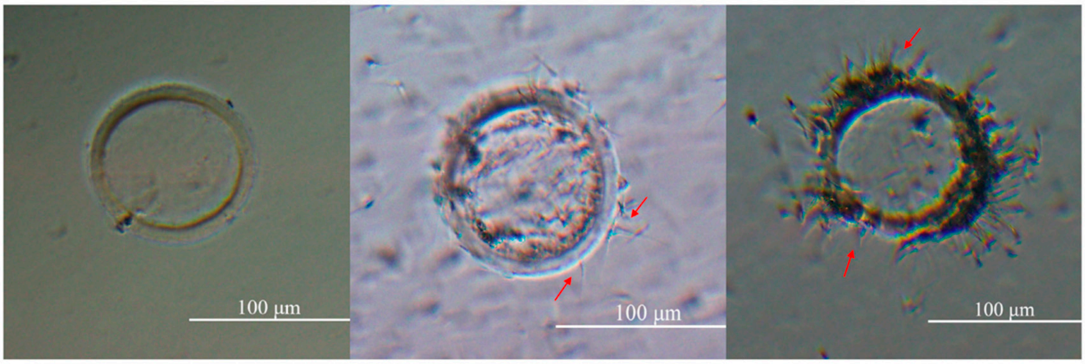

A Original appearance of zona pellucida **B** 0 nM PQQ group **C** 100 nM PQQ group
